# A YOLOv7 incorporating the Adan optimizer based corn pests identification method

**DOI:** 10.3389/fpls.2023.1174556

**Published:** 2023-06-05

**Authors:** Chong Zhang, Zhuhua Hu, Lewei Xu, Yaochi Zhao

**Affiliations:** ^1^ School of Information and Communication Engineering, State Key Laboratory of Marine Resource Utilization in South China Sea, Hainan University, Haikou, China; ^2^ School of Cyberspace Security, State Key Laboratory of Marine Resource Utilization in South China Sea, Hainan University, Haikou, China

**Keywords:** YOLOv7, smart agriculture, object detection, deep learning, pests identification

## Abstract

Major pests of corn insects include corn borer, armyworm, bollworm, aphid, and corn leaf mites. Timely and accurate detection of these pests is crucial for effective pests control and scientific decision making. However, existing methods for identification based on traditional machine learning and neural networks are limited by high model training costs and low recognition accuracy. To address these problems, we proposed a YOLOv7 maize pests identification method incorporating the Adan optimizer. First, we selected three major corn pests, corn borer, armyworm and bollworm as research objects. Then, we collected and constructed a corn pests dataset by using data augmentation to address the problem of scarce corn pests data. Second, we chose the YOLOv7 network as the detection model, and we proposed to replace the original optimizer of YOLOv7 with the Adan optimizer for its high computational cost. The Adan optimizer can efficiently sense the surrounding gradient information in advance, allowing the model to escape sharp local minima. Thus, the robustness and accuracy of the model can be improved while significantly reducing the computing power. Finally, we did ablation experiments and compared the experiments with traditional methods and other common object detection networks. Theoretical analysis and experimental result show that the model incorporating with Adan optimizer only requires 1/2-2/3 of the computing power of the original network to obtain performance beyond that of the original network. The mAP@[.5:.95] (mean Average Precision) of the improved network reaches 96.69% and the precision reaches 99.95%. Meanwhile, the mAP@[.5:.95] was improved by 2.79%-11.83% compared to the original YOLOv7 and 41.98%-60.61% compared to other common object detection models. In complex natural scenes, our proposed method is not only time-efficient and has higher recognition accuracy, reaching the level of SOTA.

## 1 Introduction

In the past decade, due to the excellent performance of machine learning and deep learning techniques on other tasks, scholars have applied them to crop pests and disease identification and have made good, progress in pests and disease identification. Scholars have applied them to crop pests and disease identification and made good progress. In 2010, Al-Bashish et al. (Al Bashish et al., 2010a). Introduced proposed the use of K-means clustering with HSI color space co-occurrence to extract color and texture features of plants, ultimately classifying five different plant diseases with a simple neural network. Since then, research works based on various machine learning methods to identify plant diseases and pests have emerged. In 2016, Sladojevic et al. (Al Bashish et al., 2010a). developed a new method for identifying 13 different plant diseases using deep convolutional neural networks, achieving a final accuracy of 96.3%. The authors created a comprehensive database and methodology for modeling, which is essential for future research in this field. Scholars have gradually realized the great potential of deep learning techniques, and research on pests and disease identification based on various deep learning methods has proliferated. For instance, Amara et al. (2017) identified and classified banana leaf diseases in the natural environment by using LeNet network, Nachtigall et al. (Bochkovskiy et al., 2020). used CNN to recognize diseases, nutrient deficiencies and herbicide damage in apple leaf images. Inspired by these previous works, our team conducted research on corn borer and anthracnose spore identification using different machine learning methods, all of which yielded promising result. However, these traditional machine learning and deep learning methods above still have their own limitations, such as high model training cost and poor robustness, which sharply increase the cost of academic research or industrial implementation. Therefore, it is important to find a pests identification method with low training cost, accurate identification and good robustness.

### 1.1 Related work and motivation

With the development of digital image processing and machine learning techniques, intelligent detection and identification of crop diseases and pests have become increasingly prevalent. In plant disease identification, Sasaki et al. (Girshick, 2015). utilized spectral reflectance differences to distinguish healthy and diseased areas on cucumber leaves, while Vízhányó et al. (Girshick et al., 2014). used color point differences to identify diseased mushrooms. In China, Guili Xu et al. (He et al., 2017). achieved over 70% accuracy in identifying tomato leaves based on histogram-based color feature extraction. Yuxia Zhao et al. (Li et al., 2022). used a Bayesian classifier to successfully identify five diseases, including maize rust. Our team has proposed several algorithms, such as the marker watershed algorithm (Lin et al., 2017a) and the Otsu separation and symbolic similarity-driven level set algorithm (Lin et al., 2017b), for accurate statistics of anthracnose spore distribution density on farms for better control. Additionally, our team proposed an accurate segmentation method for diseased fruits based on log similarity-constrained Otsu and distance rule level set activity profile evolution (Liu et al., 2019), which can achieve good segmentation of diseased fruits.

In the field of plant pests identification, various methods have been proposed to improve the accuracy and efficiency of the identification process. However, most of these methods have limitations that need to be addressed. For instance, In terms of plant pests identification, Prof. Zorui Shen of China Agricultural University (Liu et al., 2008) firstly used mathematical morphology to solve the problem and achieved good result, but the variation of the selection of structural elements in mathematical morphology will affect the identification result, then it will cause the robustness of the identification algorithm is not strong. For insects’ color characteristics, Dr. Zhu used color histogram and double-tree complex wavelet transform (Liu et al., 2016) and support vector machine (Mohanty et al., 2016) to further improve the recognition rate, but this method requires reliable data sets for training, so a large number of images need to be acquired and the cost is high. In addition, Dr. Zhu also proposed the color histogram combined with Weber descriptors for insect recognition of Lepidoptera (Nachtigall et al., 2016), CART-based combined with LLC (Redmon et al., 2016), and color-based combined with OpponentSIFT features (Redmon and Farhadi, 2017). However, these methods require manual extraction of features and are not applicable to borer moth family pests. To address these limitations, we propose an automatic pests monitoring robotic vehicle with a Pyralidae recognition scheme based on histogram and multi-template image reverse mapping method (Redmon and Farhadi, 2018). This new approach enables the automatic capture of pests images and achieves a recognition accuracy of up to 94.3% in the natural farm planting scenario. We also propose a pests image segmentation method based on GMM and DRLSE (Ren et al., 2015), which can automatically identify positive and negative samples of specific pests from a large number of scene images with recognition accuracy of up to 95%. Additionally, our proposed hybrid Gaussian model-based texture disparity representation and texture disparity-guided DRLSE model (Sammany and Medhat, 2007) can also achieve accurate segmentation of crop pests and diseases.

While the traditional machine learning methods have contributed to the field of crop pests and disease identification, they have certain limitations that prevent them from achieving the desired result. The advancements in deep learning technology have paved the way for researchers to apply deep learning algorithms to pests recognition, resulting in significant progress in this field. Deep learning algorithms can automatically extract image features, making good use of this information to achieve high accuracy in pests and disease identification. Several studies have used deep learning techniques to identify and classify pests, achieving higher robustness, generalization, and accuracy. For example, Sammany et al. (Sasaki et al., 1998). utilized genetic algorithms to improve neural networks, reducing the dimensionality of feature vectors and improving pests recognition efficiency. Similarly, Al Bashish et al. (Sladojevic et al., 2016). used the K-means clustering algorithm to classify images into clusters, extracted feature values of color and texture for each cluster, and inputted them into neural networks for classification. Mohanty et al. (Tian et al., 2019). used the GoogleNet convolutional neural network structure to build a pests identification model with satisfactory result. Compared to traditional machine learning methods, deep neural network-based pests recognition methods have better accuracy, making them an important research direction in pests recognition. As deep learning technology continues to advance, we can expect more breakthroughs in crop pests and disease identification, which will undoubtedly benefit the agriculture industry.

Deep learning models have shown promising result in identifying and detecting pests. However, there are still limitations that need to be addressed. In recent years, various sophisticated training methods have been developed to improve the generalization and robustness of deep models. Nevertheless, the cost of training these models has increased significantly due to the higher computing power required. This increase in training cost has a considerable impact on the research and industrial implementations. One common approach to reduce the training time is to increase the batch size and assist parallel training. However, a larger batch size often leads to a decrease in performance. The YOLOv7 method (Vızhányó and Felföldi, 2000), which is the current SOTA in object detection, also faces the same challenge. In this context, a new YOLOv7 corn pests identification method is proposed in this paper, which incorporates the Adan optimizer. This new method uses Adan (Wang et al., 2022), a novel optimizer that can sense the surrounding gradient information and efficiently escape from sharp local minimal areas. By replacing the original optimizer of YOLOv7 with Adan, the model can achieve faster and better training without compromising its accuracy. The proposed YOLOv7 method can identify major corn pests in complex natural environments quickly and accurately, reducing the cost of practical application of model. With fewer parameter updates, the deep model can achieve faster and more accurate identification, making it suitable for various applications. In summary, the YOLOv7 corn pests identification method incorporating the Adan optimizer presented in this paper can significantly reduce the training time and cost while maintaining the accuracy of the model. It is expected to contribute to the efficient and accurate identification of pests in agricultural production.

### 1.2 Contributions

1. To address the lack of maize pests data, we used data augmentation techniques to construct a maize pests image dataset, which effectively improved the training of the model.
2. We replaced the original optimizer of YOLOv7 with a new optimizer, Adan, which combines a rewritten Nesterov momentum algorithm with an adaptive optimization algorithm and introduces decoupled weight decay, allowing the model to increase its speed without degrading its accuracy, thus enabling faster and better training of the model and reducing the cost of implementing the model.
3. From the theoretical analysis and experimental result, it can be seen that the YOLOv7 network incorporating the Adan optimizer can effectively alleviate the negative impact caused by the increase of batch size, and solve the problem that the training speed and training accuracy cannot be achieved at the same time.

### 1.3 Paper organization

The rest of this paper is organized as follows. The second part Section 2 mainly introduces the related network model; In the third part Section 3, experimental scheme, process and results are introduced in detail; The fourth part Section 4 discusses the experimental results; The fifth part Section 5 summarizes the full text and puts forward the existing deficiencies and the direction that can be improved.

## 2 Materials and methods

This section first introduces the basic concepts of object detection network. Then it describes the YOLOv7 network and Adan optimizer used in this project, and finally introduces the proposed improved network.

### 2.1 Object detection network

**Figure 1:**
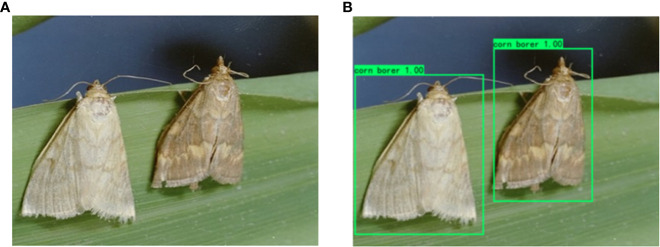
Schematic diagram of object detection: **(A)** Original map. **(B)** Object detection map.

Object detection is one of the core problems in the field of computer vision. It needs to find out all the objects of interest in an image, and determine their classes and locations. Object detection is always the most challenging problem in the field of computer vision because of the different appearances, shapes and poses of various objects, as well as the interference of illumination, occlusion and other factors during imaging. A diagram of the object detection task is shown in **Figure 1**.

The current popular algorithms can be divided into two categories, one is the two-stage algorithm based on Region Proposal, which find out some candidate regions primarily, and then adjust the regions for classification, such as the series of R-CNN (Regions with CNN features) algorithm (Xu et al., 2002; Zhao and Hu, 2015; Wang et al., 2020; Xie et al., 2022). The other category is one-stage algorithm, such as SSD (Zhao et al., 2015) (Single Shot Multibox Detector), the series of YOLO (You Only Look Once) algorithm (Vızhányó and Felföldi, 2000; Zhao et al., 2007; Zhu et al., 2015a; Zhu et al., 2015b; Zhao et al., 2019; Zhao et al., 2020), RetinaNet (Zhu et al., 2010), FCOS (Zhu et al., 2012) (Fully Convolutional One-Stage Object Detection) and other such side-to-side networks. They only use a convolutional neural network to directly predict classes and locations of different objects. Comparing the two categories of object detection algorithms, the former is more accurate but slower, while the latter is faster but less accurate. In this paper, some representative networks in the above two categories are selected for comparative experiments.

### 2.2 YOLOv7

YOLOv7 is a new network framework based on the series of YOLO algorithm, which mainly designs a better performance detection model through the following four aspects: backbone design with new ELAN module, composite model scaling, deep supervision label assignment strategy and model re-parameterization.

**Figure 2:**
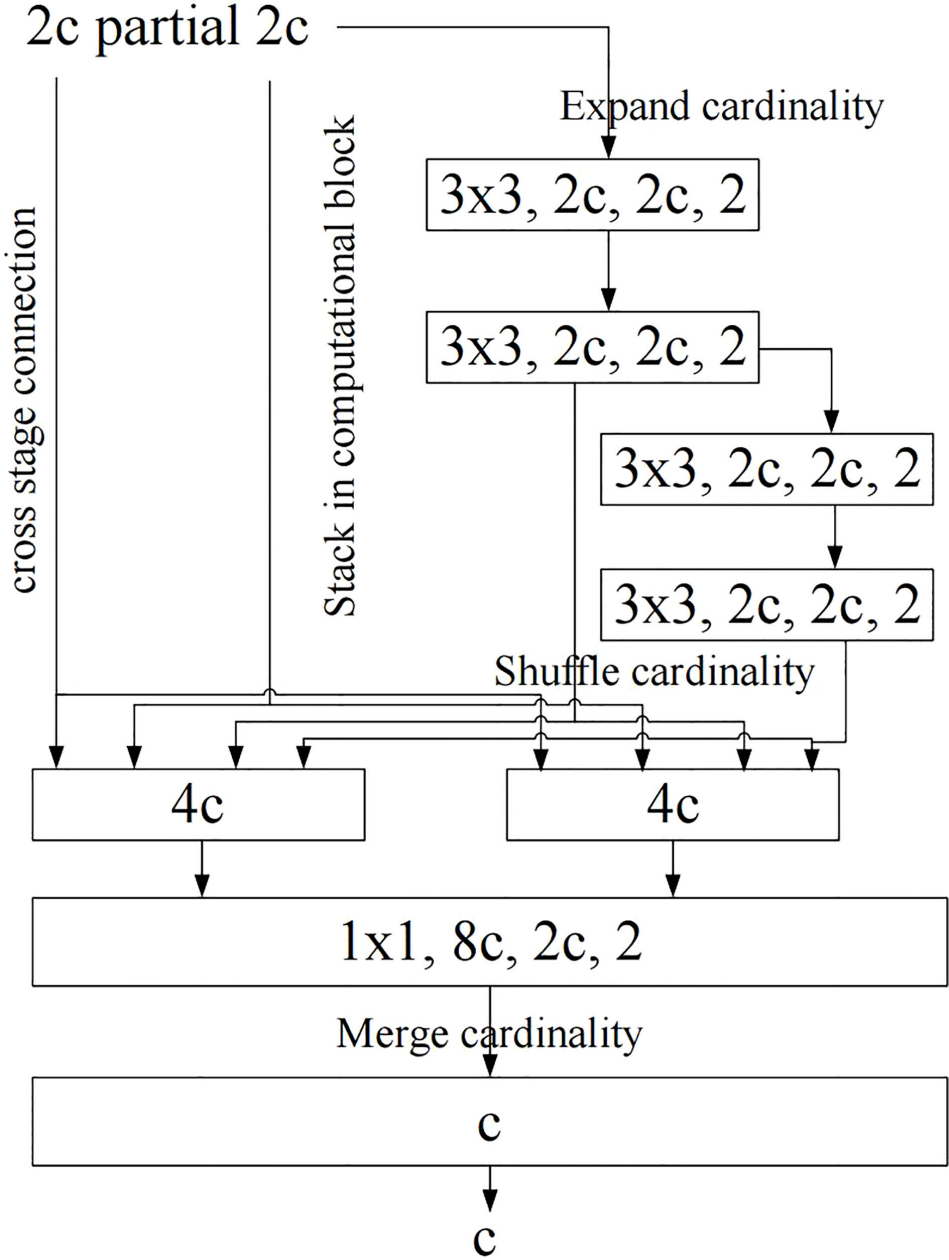
E-ELAN structure diagram. bold values means the better results.

The first improvement is the design of new network structure. YOLOv7 proposes such a view: the shortest and longest gradient paths can be controlled to achieve more effective learning and convergence of deep networks. Based on this idea, YOLOv7 designs the E-ELAN network structure as shown in **Figure 2** on the basis of ELAN. In common ELAN module, the whole network reaches a stable state regardless of the gradient path length and the number of computing modules. However, if more ELAN modules are stacked indefinitely, this stable state may be destroyed and the parameter utilization may be reduced. Based on the above shortcomings, YOLOv7 proposes the E-ELAN module. E-ELAN module adopts the structure of expand, shuffle and merge cardinality, and it can guide different computing blocks to learn more diversified characteristics compared to common ELAN module, thus improving the learning ability of the network without destroying the original gradient path.

**Figure 3:**
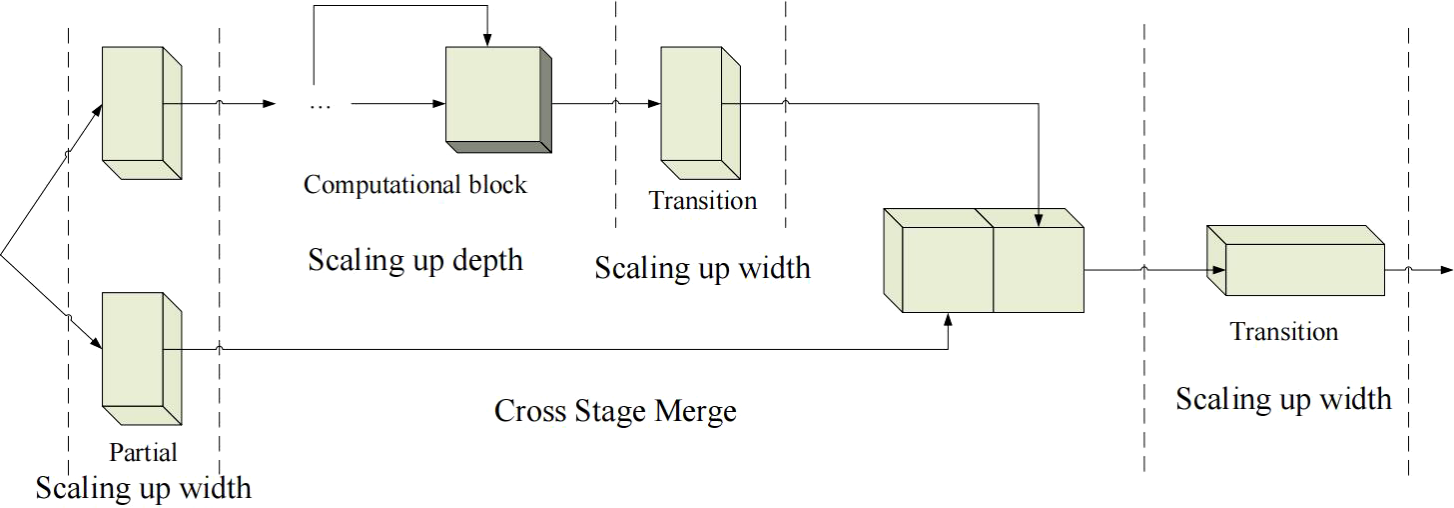
Composite model scaling for YOLOv7. bold values means the better results.

The second improvement is composite model scaling. The main purpose of model scaling is to adjust certain properties of the model and generate models of different sizes to meet the needs of different inference speeds. If the E-ELAN method described above is applied directly to a cascaded model, the action of directly scaling up the depth of the model will result in a change in the scale of the input and output channels. As a result, the model’s usage of hardware may decrease. Therefore, for the cascaded model, a composite model method must be proposed. The method must consider that the width of the transition layer should also be changed by the same amount when the depth of the computing module is scaled. Based on these ideas, YOLOv7 proposes a network architecture as shown in **Figure 3**. The network only needs to scale the depth in the computation block when performing the model scaling, and the rest of the transport block will use the corresponding width scaling. The composite scaling method can preserve the properties of the model at the initial design and maintain the optimal structure.

**Figure 4:**
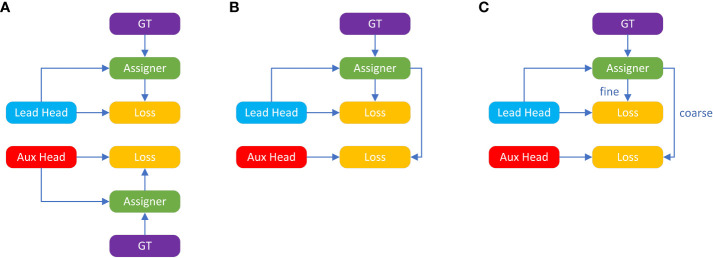
Deep supervision label assignment strategies: **(A)** Common strategy. **(B, C)** Two proposed strategies of YOLOv7. bold values means the better results.

The third improvement is deep supervision label assignment strategy. Deep supervision is a common technique in deep network training, it adds auxiliary head for loss calculation in the middle of the network to assist training. In order to differentiate auxiliary head for different functions, the final output head is called the Lead Head and the auxiliary training head is called the Aux Head. The core idea of deep supervision is to take shallow network weight and auxiliary loss as guidance, combine the output result with Ground True (GT), and use some calculation and optimization methods to generate reliable soft labels. For example, YOLO uses the bounding box regression and GT and the IOU of the prediction box as soft labels. The current common method of assigning soft labels to Aux Head and Lead Head is shown in the **Figure 4A**, which separates Aux Head and Lead Head, and uses their respective prediction result and GT to perform label assignment. In contrast, YOLOv7 network uses the Lead Head prediction result as a guide to generate coarse-to-fine hierarchical labels for Aux Heads and other Lead Heads learning. The two proposed deep supervision label assignment strategies are shown in **Figures 4B, C**. The reason for this is that the Lead Head has strong learning ability, and the generated soft labels should better represent the distribution and correlation between the source and target data. By allowing the shallow Aux Heads to directly learn the information that Lead Heads has already learned, the Lead Heads will be better able to focus on learning residual information that has not yet been learned.

The last improvement is model re-parameterization. Re-parameterization is a technique used to improve a model after training, which increases the training time but improves the inference result. Although model re-parameterization has achieved excellent performance on VGG, when applied directly to architectures such as ResNet and DenseNet, it instead causes a significant decrease in accuracy. For these reasons, YOLOv7 uses the constant connection-free RepConvN to redesign the architecture of the reparameterized convolution by replacing the 3×3 convolutional layers of the E-ELAN computational block with constant connection-free RepConv layers.

### 2.3 Adan optimizer

The most direct way to speed up the convergence of the optimizer is to import momentums. The deep model optimizers proposed in recent years all follow the same momentum paradigm used in Adam - the reball method. However, with the advent of ViT, researchers found that Adam was not able to train ViT. And AdamW, an improved version of Adam, gradually became the preferred choice for training ViT and even ConvNext. However, AdamW does not change the momentum paradigm in Adam, which tends to cause the performance of AdamW-trained networks to drop dramatically when the batch size increases to a certain threshold.

In the field of traditional convex optimization, there is an momentum algorithm equal to the heavy ball method, the Nesterov momentum algorithm. As shown in Equation 1.


(1) $$\begin{array}{c} AGD:g_{k}=\nabla f(\theta_{k}-\eta (1-\beta_{1})m_{k-1})+\xi_{k},m_{k}=(1-\beta_{1})m_{k-1}+g_{k},\theta_{k+1}=\theta_{k}-\eta m_{k} \end{array}$$

The Nesterov momentum algorithm has a faster theoretical convergence rate than the heavy ball method for smooth and generally convex problems, and can theoretically withstand larger batch size. Different from the heavy ball method, Nesterov algorithm does not calculate the gradient at the current point, but uses the momentum to find an extrapolation point, and then carries on the momentum accumulation after calculating the gradient at the point. Although Nesterov momentum algorithm has some advantages, it is rarely applied and explored in depth optimizers. One of the main reasons is that Nesterov algorithm needs to calculate gradient at extrapolated points, which requires multiple overloading of model parameters during updating at current points and requires artificial back-propagation (BP) at extrapolated points. These inconveniences greatly limit the application of Nesterov momentum algorithm in depth model optimizer.

In order to give full play to the advantages of the Nesterov momentum algorithm, Adan researchers obtained the final Adan optimizer by combining the rewritten Nesterov momentum with the adaptive optimization algorithm and introducing decoupled weight attenuation. In order to solve the problem of multiple model parameter overloads in the Nesterov momentum algorithm, the researchers first rewrote the Nesterov momentum algorithm as shown in Equation 2.


(2) $$\begin{array}{l} \begin{array}{c} \text{ Reformulated AGD}:\{\;\begin{array}{c} g_{k}=E_{\zeta ∼D}[\nabla f(\theta_{k},\zeta )]+\xi_{k}\\ m_{k}=(1-\beta_{1})m_{k-1}+[g_{k}+(1-\beta_{1})(g_{k}-g_{k-1})]\\ \theta_{k+1}=\theta_{k}-\eta m_{k} \end{array} \end{array} \end{array}$$

Combining the rewritten Nesterov momentum algorithm with the adaptive class optimizer - replacing the update of m_k from the cumulative form to the moving average form and using the second-order moment to deflate the learning rate - has resulted in a basic version of Adan’s algorithm. As shown in Equation 3.


(3) $$\text{Vanilla Adan}:\{\begin{array}{c} m_{k}=(1-\beta_{1})m_{k-1}+\beta_{1}[g_{k}+(1-\beta_{1})(g_{k}-g_{k-1})]\\ n_{k}=(1-\beta_{3})n_{k-1}+\beta_{3}{\left[g_{k}+(1-\beta_{1})(g_{k}-g_{k-1})\right]}^{2}\\ n_{k}=\eta /\sqrt{n_{k}+\epsilon }\\ \theta_{k+1}=\theta_{k}-\eta_{k}\circ m_{k} \end{array}$$

Although it can be seen that the update of m_k combines the gradient with the gradient’s difference, in real-world applications it is frequently necessary to treat the two physically distinct meaningful things separately. For this reason, the researchers developed the gradient difference momentum v_k, as shown in Equation 4.


(4) $$\begin{array}{c} m_{k}=(1-\beta_{1})m_{k-1}+\beta_{1}g_{k},v_{k}=(1-\beta_{2})v_{k-1}+\beta_{2}(g_{k}-g_{k-1}) \end{array}$$

Here different momentum/average coefficients are set for the momentum of the gradient and its difference. The gradient difference term can slow down the optimizer update when adjacent gradients are not consistent and, conversely, speed up the update when the gradients are in the same direction.

Based on the idea of L2 regular decoupling, Adan introduces a weight attenuation strategy, each iteration of Adan can be regarded as minimizing some first-order approximation of the optimization objective F, as shown in Equation 5.


(5) $$\begin{array}{c} \theta_{k+1}=\theta_{k}-\eta_{k}\circ {\bar{m}}_{k}=\underset{\theta }{\text{argmin }}(F(\theta_{k})+\langle {\bar{m}}_{k},\beta -\theta_{k}\rangle +\frac{1}{2\eta }||\theta -\theta_{k}|{|}_{\sqrt{n_{k}}}^{2}),\\ where\;||x|{|}_{\sqrt{n_{k}}}^{2}:=\langle x,\sqrt{n_{k}+\epsilon }\circ x\rangle ,\;{\bar{m}}_{k}:=m_{k}+(1-\beta_{2})v_{k} \end{array}$$

Because L2 weight regularization in F is too simple and smooth, it is unnecessary to make a first-order approximation. Therefore, only the first-order approximation of training loss can be performed and L2 weight regularization can be ignored. Then the last iteration of Adan will become as shown in Equation 6.


(6) $$\begin{array}{c} \theta_{k+1}=\theta_{k}-\eta_{k}\circ {\bar{m}}_{k}=\underset{\theta }{\text{argmin}}F(\theta_{k})+{\bar{m}}_{k},\theta -l\theta_{k}+\frac{1}{2\eta }||\theta -\theta_{k}|{|}_{\sqrt{n_{k}}}^{2} \end{array}$$

The final Adan optimization algorithm can be obtained by combining the above two improvements Equation 4 and Equation 6 into the base version of Adan.

### 2.4 The proposed identification method

**Figure 5:**
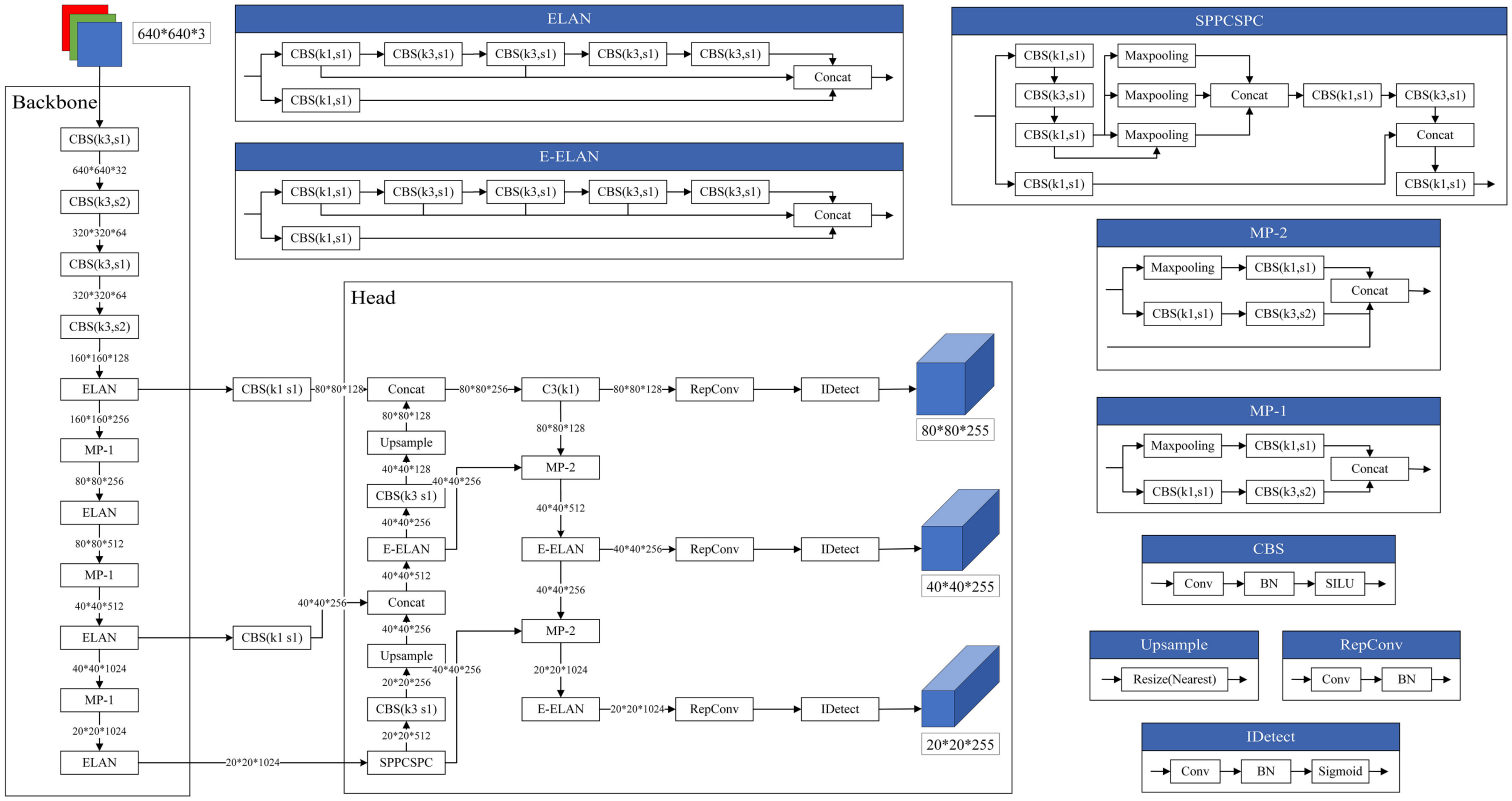
Network structure diagram.

Since the network architecture is not changed, we still use the original network structure of YOLOv7, as shown in **Figure 5**.

**Figure 6:**
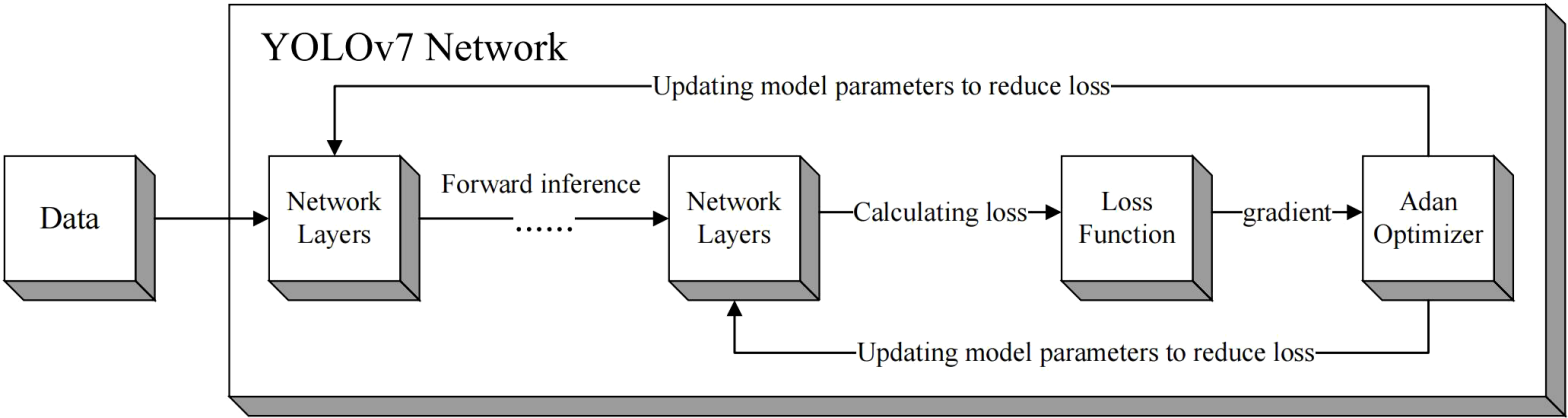
Flow chart of model training.

After replacing the optimizer inside YOLOv7 with Adan, the loss function module will calculate the loss of this forward inference according to the difference between the output of model and the real label. Subsequently, the model will take the derivative of loss to obtain the gradient of each learnable parameter. Then the Adan optimizer can obtain the gradient and update parameters through the optimization strategy described above, such as m_k, v_k, n_k, etc. The model keeps the loss decreasing by updating these parameters after each inference, thus gradually reducing the difference between the output of model and the real label, and finally achieving the convergence. The whole model training process is shown in **Figure 6**, and the pseudocode is shown in algorithm 1.

Algorithm 1 Description of the algorithm of YOLOv7 incorporating the Adan optimizer
**Input:** An image [*H*×*W*×3].
**Output:** Detection image.
**Preprocessing:** The input RGB image aligned to an RGB image of size 640×640.
**Training for** *every image in training set* **do Stage 1:** The processed images are input into the backbone module for feature extraction, while the backbone module will output three feature maps in different scales. And these feature maps will be input into the head module together for prediction.
**Stage 2:** In the head module, three types of feature maps will be fused and input into RepVGG block and detect block to predict objects.
**Stage 3:** The loss function module will calculate the loss of this inference according to the difference between the output of model and the real label. Subsequently, the model will take the derivative of loss to get the gradient and pass it to the optimizer module.
**Stage 4:** Adan Optimizer will initialize the following parameters: initialization *θ*
_0_, step size *η*, average parameter (*β*
_1_,*β*
_2_,*β*
_3_,ϵ[0,1]^3^), stable parameter, weight decays *ϵ*>0 and restart condition *λ_k_
*>0, and then start the optimizing strategy.
**for** *k*<*K* **do**
compute the stochastic gradient estimator *g_k_
* at *θ*
_k_;

$\begin{array}{l} \text{m}k=(1-\beta 1)\;mk-1+\beta 1gk/*set\;m0=g0*/\\ \text{v}k=(1-\beta 2)\;vk-1+\beta 2(gk-gk-1)/*set\;v1=g1-g0*/;\\ \text{n}k=(1-\beta 3)\;nk-1+\beta 3{\left[gk+(1-\beta 2)(gk-gk-1)\right]}^{2}\\ \text{n}k=\eta /(\sqrt{nk+\epsilon })\\ \theta k+1=(1+\lambda k\eta )-1[\theta k-\eta k^{-1}\circ (mk+(1-\beta 2)vk)] \end{array}$

**if** *restart condition holds* **then**
get stochastic gradient estimator *g*
_0_ at *θ*
_k+1_;
*m*
_0_ = g_0_, *v*
_0 =_ 0, *n*
_0_ = (*g*
_0_) ^2^, update *θ*
_1_ by Line 7, *k* = 1;
**end
end
end**

## 3 Experiments and result

### 3.1 Experimental scheme

**Figure 7:**
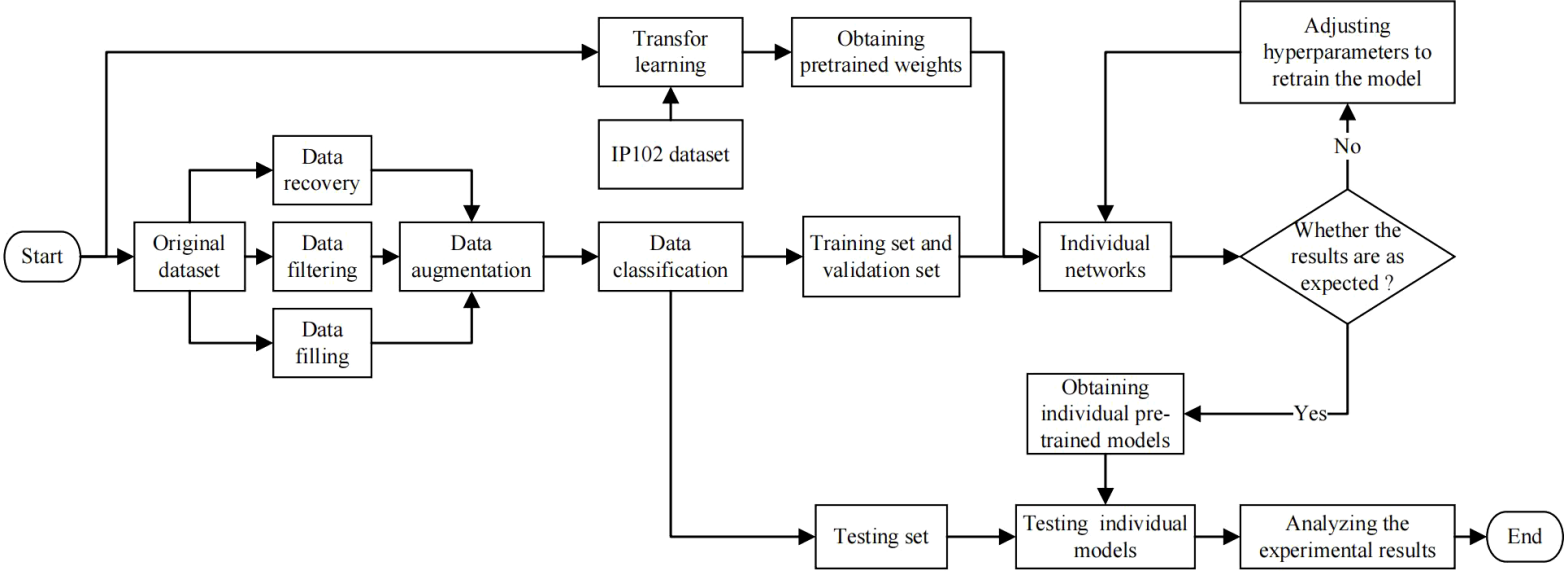
Flow chart of experimental scheme.

The experimental scheme proposed is shown as **Figure 7**. We first pre-processed the original dataset, mainly including data recovery, data filtering and data filling. In order to solve the problem of scarce data, we used data augmentation and transfer learning to ensure that the network can fully learn the features. The two technologies will be introduced in detail in the following sections. And then, the augmented dataset was divided into training set, testing set and validation set. The training set and validation set was input into the original YOLOv7 network, the improved YOLOv7 network and other comparative networks respectively. If the performance of the model does not meet expectations, we will adjust the network’s hyperparameters and retrain it. After that, the testing set was input into trained models to test the performance of different models. Finally, we compared and analyzed the experimental result.

### 3.2 Evaluation metrics

For binary classification problem, A is called “positive” and B is called “negative,” and the classifier correctly predicts “True” and incorrectly predicts “False”. According to these four basic combinations, the four basic elements of the confusion matrix are TP (True Positive), FN (False Negative), TN ((True Negative), and FP (False Positive), as shown in **Table 1**.

**Table 1:** Confusion matrix.

Truth	Prediction	
	T	F

P	TP	FN

N	FP	TN


In object detection experiments, IoU, Precision, Recall, AP and mAP are commonly used as evaluation indexes. Among them, IoU represents the intersection ratio between the predicted result and the true label for each category, as shown in Eq.7. Precision refers to the proportion of data whose value is true indeed when the classifier predicts it to be true, while Recall refers to the percentage of the classifier predicts to be correct for all data that is true, respectively, the formulas of the two is Eq.8 and Eq.9. However, all three indexes have their limitations, therefore AP/mAP is often used to evaluate the performance of object detection task.


(7) $$\begin{array}{c} \text{ IoU }=\frac{TP}{TP+FP+FN} \end{array}$$

(8) $$\begin{array}{c} \text{ Precision }=\frac{TP}{TP+FP} \end{array}$$

(9) $$\begin{array}{c} \text{ Recall }=\frac{TP}{TP+FN} \end{array}$$

**Figure 8:**
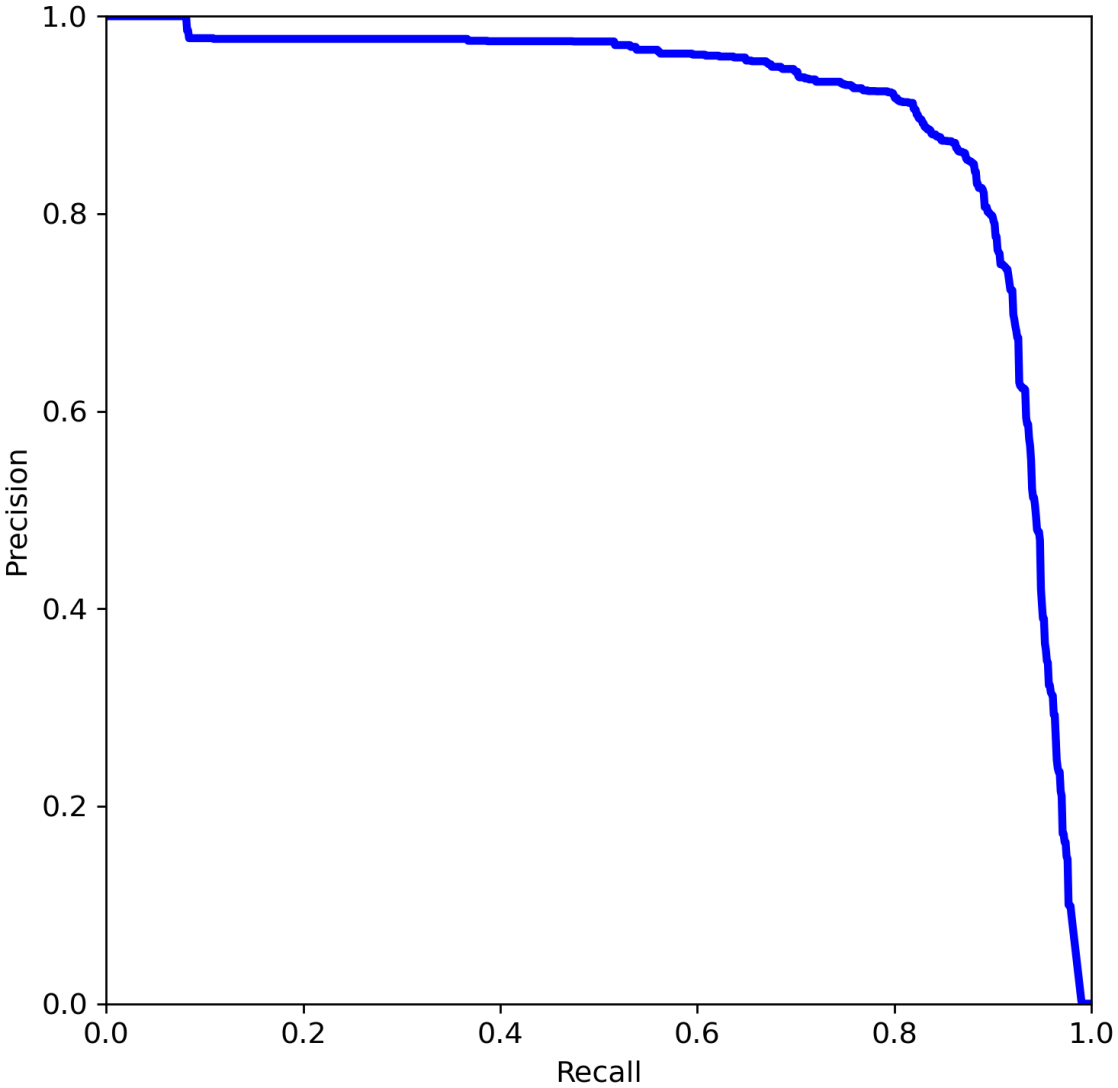
Schematic diagram of PR curve.

If we take different confidence levels, we can get different Precision and Recall, and if we get the confidence level dense enough, we will obtain the Precision-Recall curve(PR curve), as shown in **Figure 8**. While AP refers to the area under the curve, and mAP is the average of the AP values for all classes. In particular, the mAP@[.5:.95] refers to the mAP at different IoU thresholds (from 0.5 to 0.95, in steps of 0.05).

### 3.3 Dataset acquisition

Due to the scarcity of public corn pests dataset, we collected some images of three major corn pests: corn borer, bollworm, and armyworm on the web as our original dataset, including 31 images of corn borer, 36 images of bollworm, 31 images of armyworm and 31 negative images. Prior to beginning the experiment, we used data augmentation techniques to the technique expands a total of 129 images to 5160 images as our final dataset. During training, we use a ratio of 8:1:1 to split the dataset into a training set, a validation set and a testing set. And the training set has 4128 images, the validation set has 516 images and the testing set has 516 images.

### 3.4 Data augmentation

**Figure 9:**
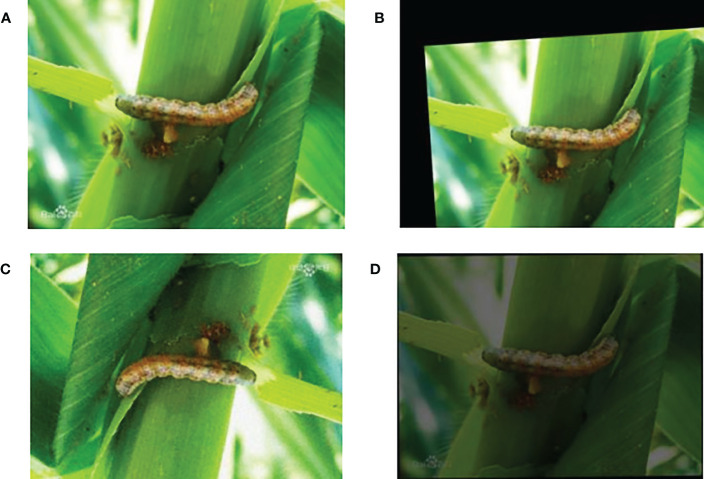
Data augmentation effect display: **(A)** Original image. **(B)** Random crop. **(C)** Flip. **(D)** Decrease in brightness.

As deep learning requires a large amount of data for training, we used data augmentation on the original dataset, such as random rotation transform, blur transformation, flip transform, addition of Gaussian noise and so on. The random rotation and flip transformation models are able to simulate the different locations of insect presence, while the blur transformation and Gaussian noise could better simulate the various environment that may occur in reality. **Figure 9** shows the images which performing data augmentation.

### 3.5 Transfer learning

Transfer learning is a popular method in the field of computer vision, because it can build accurate models in less time. By using transfer learning, model do not start training from scratch, but start with the patterns of solving problems that learned from previous problems. In the field of computer vision, transfer learning is usually represented by the use of pre-trained models. Pre-trained models are models that trained on large baseline datasets. For example, in object detection tasks, backbone neural network is first used for feature extraction. The backbone used here is generally a neural network such as VGG, ResNet, etc. Therefore, when training an object detection model, the parameters of the backbone can be initialized by using the pre-trained weights of these neural networks so that more effective features can be extracted at the beginning.

In this paper, we selected the IP102 public dataset as a pre-trained dataset^1^. The IP102 dataset is a large-scale dataset for pests identification, which contains more than 75,000 images of 102 pests classes. These images exhibit a natural long-tailed distribution. In addition, about 19,000 of these images have added bounding boxes for object detection. We select these images with object detection frames, and feed them into individual networks for training to obtain pre-trained weights. The pre-trained weights will be transferred to our own dataset for use, and it can make the final model more robust and convincing in the pests identification task.

### 3.6 Experimental environment and parameter settings

The experimental environment configuration of this paper is as follows: OS is Linux, GPUs are two Tesla V100 with 80G memories, training environment is python 3.7, Pytorch 1.11.0. while Labelme is used to annotate the data. In training, to ensure comparability across experiments and appropriateness of training, each training epoch consists of 100 rounds and the img_size is 320×320. In order to verify the good performance of our proposed algorithm under large batch size, we set the batch size to 512. While for training of YOLOv7, the weight_decay is 0.002 and learning rate is 0.001.

### 3.7 Experiment result

**Figure 10:**
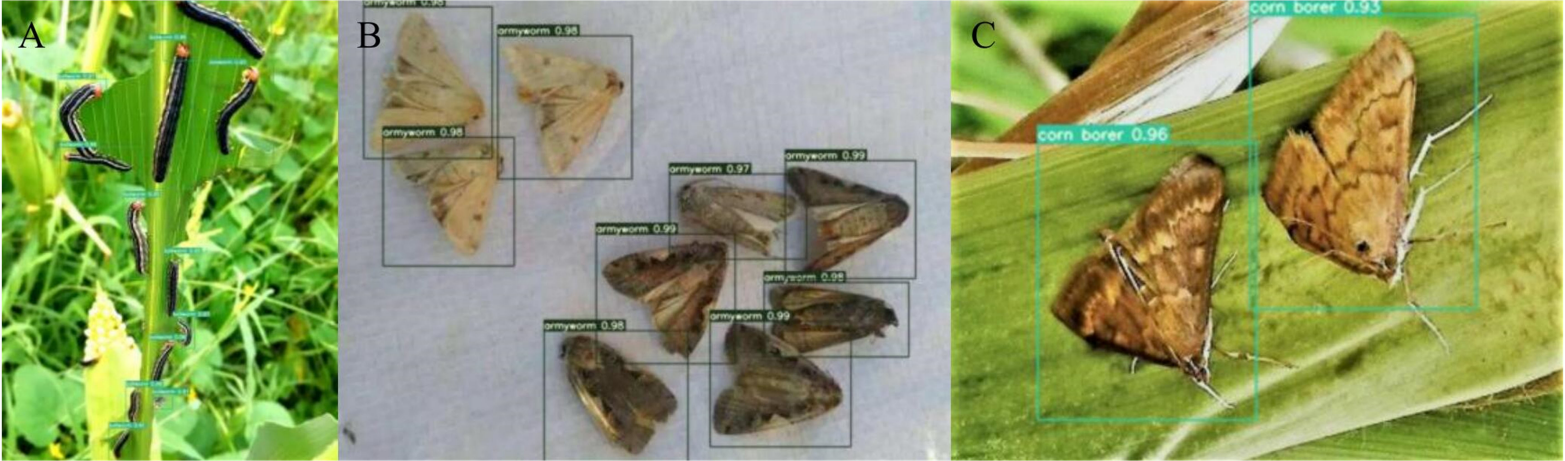
Test result: **(A)** bollworm. **(B)** armyworm. **(C)** corn borer.

**Figure 10** shows the prediction performance of the YOLOv7 network incorporating with the Adan optimizer when facing different species of maize pests.

In order to verify the effectiveness of the algorithm proposed in this paper, we compared the improved network with the original network which using Adam, AdamW and SGD. We also tested several other object detection networks: SSD (Zhao et al., 2015), RetinaNet (Zhu et al., 2010), FCOS (Zhu et al., 2012), Faster RCNN (Xu et al., 2002) and FPN (Zhu and Zhang, 2013). Finally, we put these networks together and compared them with the result of our previous works, and the performance evaluation indexes are [mAP@.5:.95] and precision which are described above. The result is shown in **Table 2**.

**Table 2:** Performance comparison of different networks.

Networks	mAP@[.5:.95]	Precision
YOLOv7(Adam) (Vızhányó and Felföldi, 2000)	0.8646	99.66%
YOLOv7(AdamW) (Vızhányó and Felföldi, 2000)	0.9032	99.64%
YOLOv7(SGD) (Vızhányó and Felföldi, 2000)	0.9407	99.91%
Faster R-CNN (Xu et al., 2002)	0.6655	88.99%
SSD (Zhao et al., 2015)	0.6608	98.87%
RetinaNet (Zhu et al., 2010)	0.681	98.2%
FCOS (Zhu et al., 2012)	0.602	86.8%
FPN (Zhu and Zhang, 2013)	0.6337	87.6%
Histogram Reverse Mapping and Invariant Moment (Redmon and Farhadi, 2018)	None	94.3%
GMM and DRLSE (Ren et al., 2015)	None	86.364%
Ours	**0.9669**	**99.95%**

The bold values means the better results.

**Figure 11:**
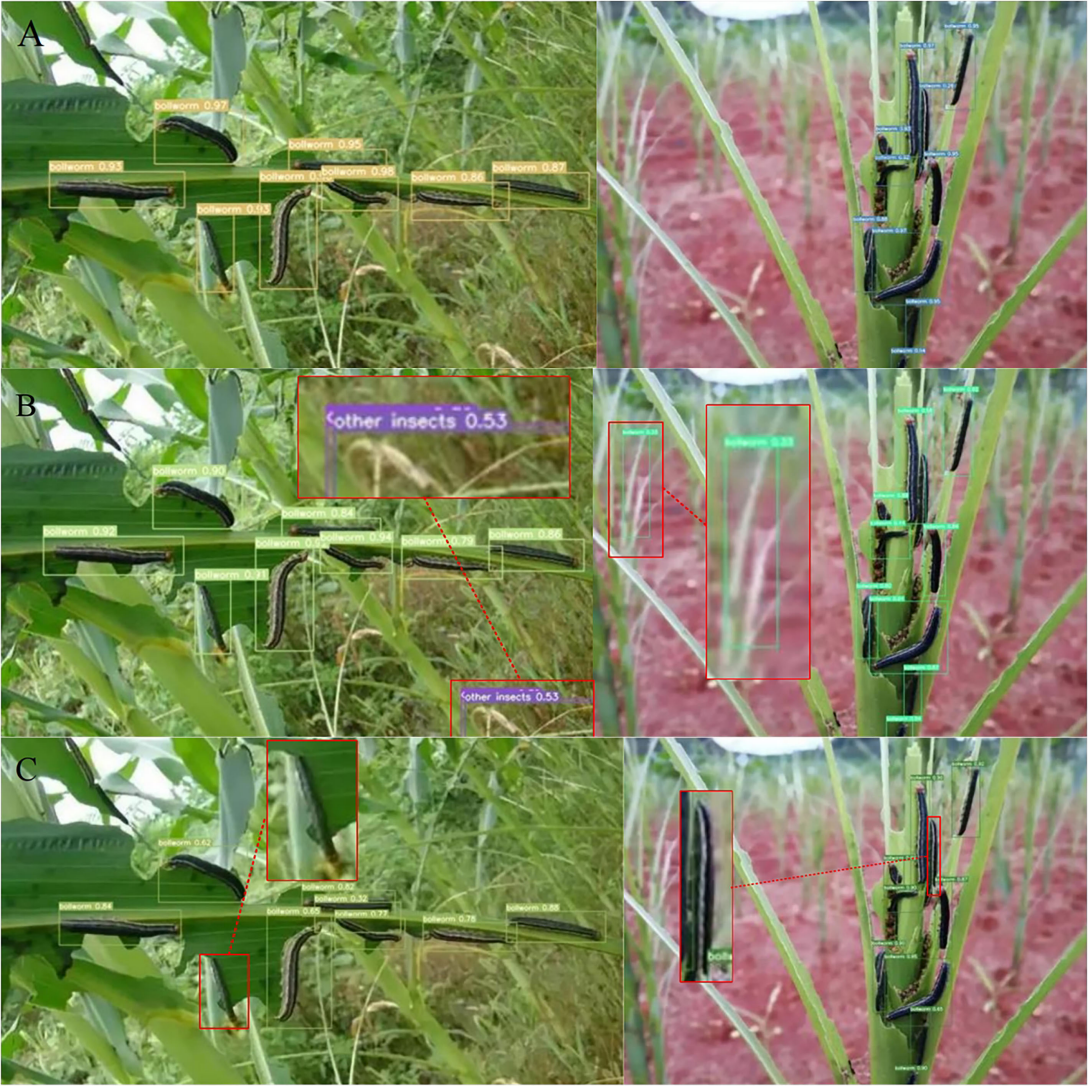
Comparison of the prediction effect of different networks when facing the same image: **(A)** YOLOv7(Adan). **(B)** YOLOv7(Adam). **(C)** SSD.

We also compared the differences between the YOLOv7 network loaded with Adan and other networks when face with the same image. And the prediction result are shown in **Figure 11**.

## 4 Discussion

**Figure 12:**
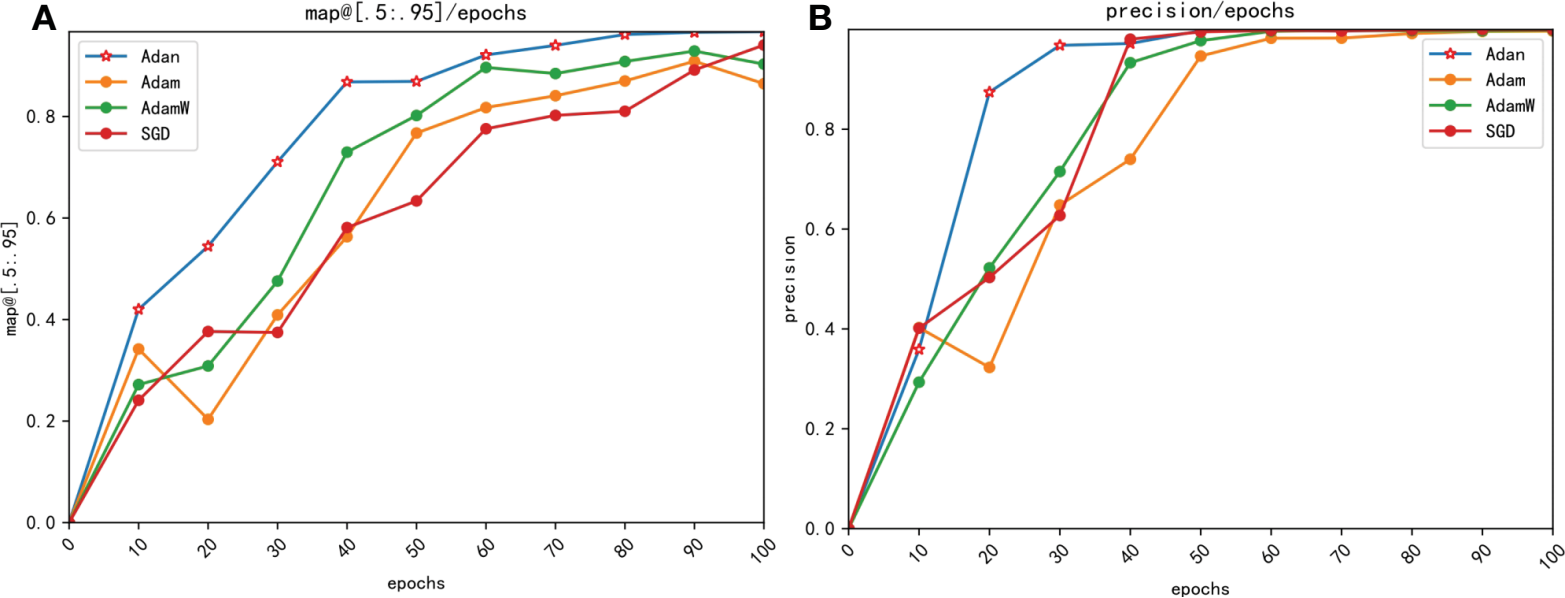
Comparison of precision changes with epoches for different optimizers. **(A)** map@[.5:.95] **(B)** precision.

The experimental result in **Table 2** shows that the YOLOv7 network incorporating the Adan outperforms traditional ML algorithms and other comparative networks in the comparison of both mAP@[.5:.95] and precision. Meanwhile, from **Figure 10** we can see that the improved network has a good performance on different types of maize pests. What’s more, further comparison of three different networks in **Figure 11** shows that the YOLOv7 network incorporating Adan can still perform well in more complex natural environment with no errors. SSD network and the YOLOv7 network incorporating the Adam both have errors in prediction of the same images. The YOLOv7 network with the Adam misidentified the background as insects in two images, while SSD network misidentified insects as the background in both images. The final comparison of performance indexes and prediction result verifies that Adan optimizer can effectively improve the model performance and help the YOLOv7 network reduce the possibility of false recognition and missed recognition, thus making the network more efficient and error-free in pests recognition task. For further confirmation, we collected data of the map[.5:.95] and precision of YOLOv7 networks which using different optimizers in the experiment when the epoch changed, as shown in **Tables 3**, **4**. Based on these data, we plotted the performance trends of four optimizers, as shown in **Figure 12**.

**Table 3:** Comparison of [mAP@.5:.95] changes with epochs for different optimizers.

epochs	Ours	YOLOv7(Adam)	YOLOv7(AdamW)	YOLOv7(SGD)
10/100	**0.4202**	0.342	0.2719	0.241

20/100	**0.5444**	0.2034	0.3086	0.3765

30/100	**0.7107**	0.4091	0.4758	0.3746

40/100	**0.8681**	0.5632	0.7297	0.5812

50/100	**0.8691**	0.7674	0.802	0.6337

60/100	**0.9212**	0.8176	0.8966	0.7758

70/100	**0.9401**	0.8409	0.8846	0.8022

80/100	**0.9615**	0.8699	0.9083	0.8103

90/100	**0.9658**	0.9089	0.9289	0.8916

100/100	**0.9669**	0.8646	0.9032	0.9407


The bold values means the better results.

**Table 4:** Comparison of precision changes with epochs for different optimizers.

epochs	Ours	YOLOv7(Adam)	YOLOv7(AdamW)	YOLOv7(SGD)
10/100	0.3589	**0.4026**	0.2933	0.4015

20/100	**0.8744**	0.3229	0.5222	0.5031

30/100	**0.968**	0.6481	0.7153	0.6272

40/100	0.9717	0.7398	0.9332	**0.9803**

50/100	**0.9974**	0.9466	0.9774	0.995

60/100	**0.9981**	0.9823	0.9962	0.9979

70/100	**0.9986**	0.9825	0.9978	0.9967

80/100	**0.9991**	0.9918	0.9972	0.9989

90/100	0.999	0.9959	0.997	**0.9997**

100/100	**0.9995**	0.9966	0.9982	0.9991


The bold values means the better results.

From **Figure 12** we can see that the YOLOv7 incorporating with Adan optimizer converges faster than YOLOv7 loaded with other optimizers in both mAP@[.5:.95] and precision, and the result are consistent with our theoretical analysis. In process of calculating momentums, Adan uses the modified Nesterov momentum algorithm, while Adam with AdamW use the traditional reballing algorithm. The modified Nesterov momentum algorithm helps Adan to sense the surrounding gradient information in advance, which helps model to escape from the sharp local minimal regions efficiently, thus speeding up the convergence of Adan. The comparison of map[.5:.95] and precision shows that Adan can obtain greater performance by using only 1/2-2/3 of the computation of other optimizers. What’s more, the mAP@[.5:.95] increases by 2.79%-11.83% compared to original optimizers. The experimental result also confirm that Adan only needs less than 2/3 of computation of the original network to obtain the performance beyond it, which is proposed in the original paper of Adan.

## 5 Conclusions

In this paper, a new deep learning algorithm based on YOLOv7 network and Adan optimizer is proposed, and a feasible maize pests identification scheme is proposed as well, which is successfully applied to the identification task of maize pests. The mAP@[.5:.95] of the improved network reaches 96.69% and the precision reaches 99.95% in this task, which breaks the bottleneck of the original networks. And it also confirms the feasibility and effectiveness of applying deep convolutional neural networks to the task of crop pests and disease identification, and it has positive significance for crop pests and disease prevention and control. We can quickly identify common corn pests and take appropriate measures by using this model, and scientifically carryout pests control methods to reduce possible economic losses and promote agricultural modernization.

However, the environment is more complex in real life. There are many other insects with similar characteristics, while the difficulty of detection in complex environment will be greatly increased due to the limitations of scarce data. Meanwhile, some corn pests will appear in the form of eggs in real life, while these eggs are tiny and their characteristics are difficult to distinguish, making identification more difficult. What’s worse, pests data are scarce and difficult to collect, and the cost of manual labeling is very high. Therefore, how to obtain sufficient data and enough computing power is the key of future pests controlling technology researches.

## Data availability statement

The original contributions presented in the study are included in the article/supplementary material. Further inquiries can be directed to the corresponding author.

## Author contributions

Conceptualization, ZH, YZ and CZ; methodology, CZ and LW; software, CZ and LW; validation, ZH, CZ, and LW; formal analysis, ZH, LW, and CZ; investigation, ZH and YZ; resources, ZH; data curation, CZ and LW; writing—original draft preparation, CZ and LW; writing—review and editing, ZH; visualization, CZ; supervision, ZH; project administration, ZH and YZ; funding acquisition, ZH and YZ. All authors contributed to the article and approved the submitted version.

## Footnotes

1
https://github.com/xpwu95/IP102
